# The noncoding universe

**DOI:** 10.1186/1741-7007-9-52

**Published:** 2011-07-28

**Authors:** Kester Jarvis, Miranda Robertson

**Affiliations:** 1 BMC Biology, BioMed Central Ltd, Floor 6, 236 Gray's Inn Road, London, WC1X 8HB, UK

## Editorial

The 'dark matter' of cosmologists has little in common with the so-called dark matter of the genome other, it would seem, than the controversy that surrounds both. Whereas cosmological dark matter was inferred from gravitational effects that cannot be explained by known bodies in the universe, genomic dark matter emerged from the application of post-genomic technology to the analysis of the transcriptome, and could not have been inferred from any known biological principle. In biology, the term is (somewhat romantically) applied to the surprisingly extensive transcription of RNA from regions of the genome which do not code for proteins; and whereas there is still no firm evidence that cosmological dark matter exists, never mind any evidence on what it is, the existence of noncoding RNA is not in dispute. The question is how much there is of it, and what it means.

## The answer is yes

*BMC Biology *first tackled these questions [1] on the publication of a somewhat combative paper from Tim Hughes and colleagues in *PLoS Biology *[2] challenging earlier claims of pervasive transcription of noncoding DNA, and of a Q&A for *BMC Biology *from John Mattick, who is a particularly energetic and articulate exponent of the notion that an extensive noncoding transcriptome with regulatory functions accounts for the evolution of organismal complexity [3]. More than a year on, *PLoS Biology *has published a rejoinder from Mattick and colleagues [4] to the paper from van Bakel *et al. *[2], along with a rejoinder to the rejoinder from van Bakel *et al*.[5].

The original contention of van Bakel *et al. *was, broadly speaking, that if you analyze the transcriptome with RNA-seq technology rather than with the tiling array technology that is the basis for much of the most notable evidence of pervasive transcription, the genome looks considerably less pervasively transcribed; and that most of the noncoding transcription occurs in the vicinity of active genes and can probably be accounted for by leaky transcriptional machinery and excised introns. One obvious question - raised at the time, and now explored by Clark *et al. *[4] - is whether RNA-seq analysis is more reliable than microarray-based techniques for the measurement of the transcriptome. The more fundamental and difficult question is whether most of the noncoding RNA of unknown function is in fact just transcriptional noise and meaningless intronic debris, or whether it has important regulatory functions yet to be discovered. These questions are not answered by the opposing Perspective articles published in *PLoS Biology*.

The following statements can probably be regarded as non-contentious. Most dark matter transcripts are, as stated in the title of van Bakel *et al. *[2] and acknowledged by Clark *et al. *[4], associated with known genes. Rare transcripts that are detected by microarray analysis may be missed by RNA-seq. But in general, when the two methods are directly compared, they give comparable answers [4,6,7]. Indeed, we could argue that there is remarkably little disagreement on major points of fact, the main difference being in the positions taken by the principal protagonists in the pervasive transcription debate. For example, Kapranov and colleagues [8], who argue vigorously for the pervasiveness and potential importance of dark matter transcription, studied the transcriptome using a single-molecule sequencing method that removes as many sample preparation steps - and consequent bias in the results - as possible, and arrived at an estimate for the proportion of exonic transcripts that is essentially comparable to that just published by van Bakel and colleagues — around 40-50% — with the remainder being intronic and intergenic dark matter.

## But what's the question?

The debate continues, however, despite the scope for consensus, and the sticking point remains the same: reasonable arguments can be mustered for rare ncRNA transcripts' being either functional or artefactual, whatever the method used to determine how many of them there are. Many, very reasonably, take the view that it is time to stop arguing about the content and implications of the transcriptome and refocus on finding evidence for dark matter functions. This impeccable aim may however require considerable ingenuity to achieve. As Ponting and Belgard have pointed out [9], established tests of function that depend on gene knockout or overexpression only work for a fraction even of known protein-coding genes. More subtle means of interrogation may have to be devised for extracting the uncharted functions of the noncoding transcriptome.
